# Acoustic emission-based intelligent identification of piston aero-engine ignition advance angle anomalies

**DOI:** 10.1038/s41598-023-44755-7

**Published:** 2023-10-20

**Authors:** Yanhe Yang, Xiaoyang Bi, Alamusi Lee, Teng Ma, Yinghui Sun, Wei Kong, Wei Hu, Ning Hu

**Affiliations:** 1 School of Mechanical Engineering, Hebei University of Technology, Tianjin, 300401 People’s Republic of China; 2 State Key Laboratory of Reliability and Intelligence Electrical Equipment, Hebei University of Technology, Tianjin, 300401 People’s Republic of China; 3 Xinjiang Tianfu Energy, East 2 Bei Yi Road, Shihezi, 832000 Xinjiang People’s Republic of China; 4 World Transmission Technology (Tianjin), Intersection of Gaoxin Avenue and Jingming Road, Beichen Science Park, Tianjin, 300130 People’s Republic of China

**Keywords:** Engineering, Mechanical engineering

## Abstract

Ignition advance angle is one of the important factors affecting the performance of the engine, when it occurs abnormally will make the engine power and economy worse, and even cause serious damage to the engine. Therefore, it is very necessary to recognize the abnormal ignition advance angle of the engine. However, the engine system is closed and has a complex structure, which makes traditional diagnostic methods difficult. This paper proposes an intelligent identification method based on acoustic emission (AE) signals, which collects the AE signals from the engine surface and divides their spectra into equal parts, and selects the frequency bands with high contribution to the classification based on the minimum distance method to construct feature maps, which is used as the input to the convolutional neural network (CNN). The extracted frequency band features of this method can better characterize the AE signals, and the constructed feature maps make the fault information more obvious. Experiments show that the accuracy of this method for abnormal ignition advance angle under normal operating conditions of piston aero-engine is 100%, which is better than the traditional methods. In addition, the recognition accuracies under the other two operating conditions are 99.75% and 98.5%, respectively, indicating that the method has a certain universality.

## Introduction

**Figure 1:**
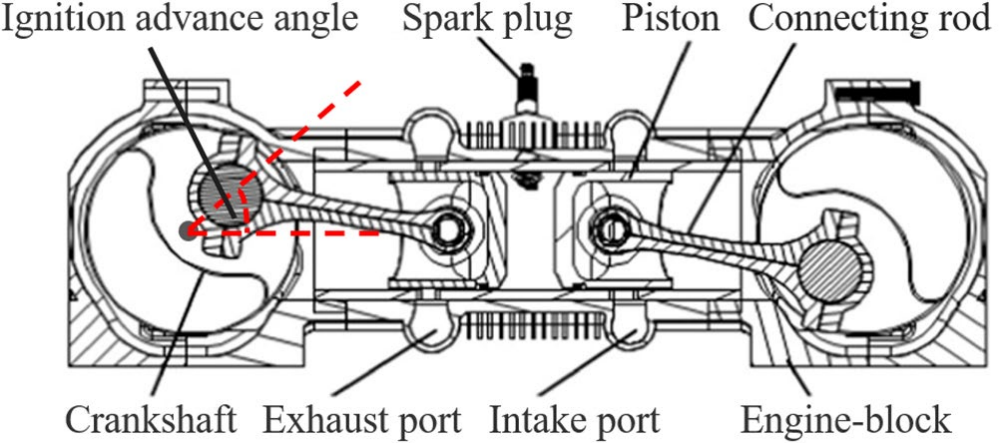
Sectional view of the internal structure of the engine^2^.

Piston aero-engines are widely used as the core power unit of low-speed light aircraft because of their small size, light mass, low fuel consumption and high power-to-weight ratio, and their performance directly affects the operational safety and economic efficiency^1^. Ignition advance angle, as one of the main operating parameters of the engine, and refers to the angle at which the crankshaft turns from the ignition of the spark plug to the point at which the piston reaches the upper stop of compression, as shown in Fig. 1. The ignition advance angle is related to the engine speed, load, air temperature, and fuel quality, etc. Each engine condition corresponds to an optimal ignition advance angle, so the reasonableness of its setting has an important impact on the engine’s power, fuel consumption rate, and emission performance, due to the size of which is usually automatically adjusted by the engine control unit (ECU) or manually adjusted by human beings, the abnormalities of the ignition advance angle are often occurring. Abnormal ignition advance angle will not only reduce the engine performance and deteriorate the fuel economy, but also lead to strong engine detonation, which is a threat to the normal operation of the engine, thus posing a great safety hazard.

In the past few years, many scholars have made extensive research on the influence of ignition advance angle on engine performance. Jacek et al. found that the magnitude of the ignition advance angle has a significant impact on the combustion process in the engine cylinder by analyzing the cylinder pressure signal^3^. If the ignition advance angle is too large, the maximum combustion pressure in the cylinder will be reached before the piston reaches the top dead center; if the ignition advance angle is too small, the mixture in the cylinder will not be fully burned until the piston travels down a long distance. Therefore, when the ignition advance angle is abnormal, whether it is too large or too small, it will cause the power of the engine to decrease and the fuel consumption to increase. Li et al. optimized the fuel injection advance angle and ignition advance angle, and found that the optimized braking ratio and fuel consumption ratio were both increased by more than 10% compared with the non-optimized condition^4^. Mehra et al. and Li et al. found that the ignition advance angle not only affects the performance of the engine, but also is related to the emission of harmful gases such as CO and NOx, and it is experimentally concluded that the ignition advance angle is positively correlated with the emissions^5,6^. Not only that, many studies have shown that the ignition advance angle has a great impact on engine knock^7–9^. Ma et al. found that with the increase of the ignition advance angle, the pressure and temperature in the cylinder rise rapidly, which increases the possibility of engine knock combustion^2^. Although a slight knock is beneficial to the performance of the engine, if it is uncontrolled and develops into a strong knock, it will reduce the performance of the engine and even cause serious damage to the engine structure. Therefore, in order to avoid the occurrence of the above events, it is of great significance to identify and study the abnormal state of engine ignition advance angle.

In previous cases of engine fault identification, the analysis of fault features using vibration signals is often considered as one of the most effective diagnostic tools^10,11^. Compared with vibration signals, acoustic emission (AE) is a non-destructive testing technology that was mostly used to detect early damage within materials initially^12,13^. Due to the excellent properties of AE signals such as wide signal frequency domain, rich information, high signal-to-noise ratio, and sensitivity to early weak faults, they have been widely used in many fields today^14,15^. In terms of engine fault diagnosis, Elamin et al. used the time domain and frequency domain information of the AE signal to accurately identify the valve fault of the engine^16^. Bejger et al. realized the status monitoring of the fuel injection pump by analyzing the AE signal of the marine diesel engine^17^. Han et al. used the AE signal to identify the early weak damage of the engine structure^18^. This shows the potential of using AE signals to diagnose engine faults. In addition, the AE signal is very sensitive to the impact of high-pressure gas. The combustion explosion pressure in the cylinder will excite the AE signal on the surface of the cylinder body, and the ignition advance angle significantly affects the combustion process in the cylinder. Therefore, the abnormality of the ignition advance angle will make the AE signal changes of the cylinder during the combustion phase, so we use the AE signal to identify the abnormality of the ignition advance angle has certain advantages. To the best of our knowledge, the existing literature does not utilize the AE signal to identify the engine ignition advance angle abnormality, which is one of the innovations of this paper.

Traditional AE signal processing methods mainly include parameter analysis^19^ and waveform analysis^20^. Due to the blindness of human extracted features, redundant features with low or no contribution to fault classification often appear in our extracted features, which will not only affect the computational efficiency of the model but also reduce the recognition accuracy. Therefore, we need to perform the necessary feature selection^21,22^. Among the common feature selection methods, the embedded method is easily affected by the performance of the classifier, the wrapper method has high computational complexity and poor performance in high dimensional space, in addition, the filter method usually uses the distance to evaluate the separability of the categories, which is the most commonly used, but the computational process is cumbersome^23–25^. This paper is also based on the distance separability, by comparing the minimum value of the distance between feature classes for feature selection, the method significantly simplifies the calculation process under the premise of ensuring high classification ability.

As an end-to-end classification algorithm, deep learning not only eliminates the tedious process of traditional manual feature extraction, but also has high classification accuracy, so it is widely used in many fields including mechanical equipment fault diagnosis^26–28^. In terms of engine fault diagnosis, Hosseini et al. accurately identified engine knock based on a deep neural network (DNN)^29^. Li et al. used the deep echo state network (DESN) to complete the diagnosis of engine fuel injection failure and valve failure^30^. Gou et al. combined the continuous wavelet transform and CNN method to identify engine sensor faults with remarkable results^31^. Compared with other deep neural networks, CNN can reduce the computational effort by sharing the convolutional kernel parameters while ensuring the same functionality or accuracy. Therefore, CNN can be well used to fit data-intensive acoustic emission signals.

In this paper, a method is proposed to identify the ignition advance angle anomalies of piston aero-engine by combining the advantages of AE signal and CNN model. The final experimental results show that the method has better recognition accuracy and recognition efficiency. The main contributions are as follows:

1. Based on the excellent features of the AE signal, the AE signal is introduced into the abnormal recognition of the engine ignition advance angle as the acquired information.
2. Aiming at the problem of huge amount of AE signal data, a feature extraction method is proposed: the spectrum of the signal is divided into equal parts, and the maximum value of the amplitude on each frequency band is used as the feature of the segment, and the frequency bands with large contribution are screened using a distance-based method to obtain the feature vector. Finally, the feature map is constructed as the input of the CNN.
3. A series of experiments were carried out and compared with different methods. The experimental results show that the proposed method can significantly improve the recognition accuracy and recognition efficiency and has good universality for other working conditions.

The paper is structured as follows: “Introduction” section introduces the background and significance of the study. “Methodology” section describes the theoretical knowledge and the proposed method used in this paper. “Experimental verification” section establishes the experiments to verify the effectiveness of the proposed method. Finally, the conclusion and outlook are given in “Conclusion and outlook” section.

## Methodology

**Figure 2:**
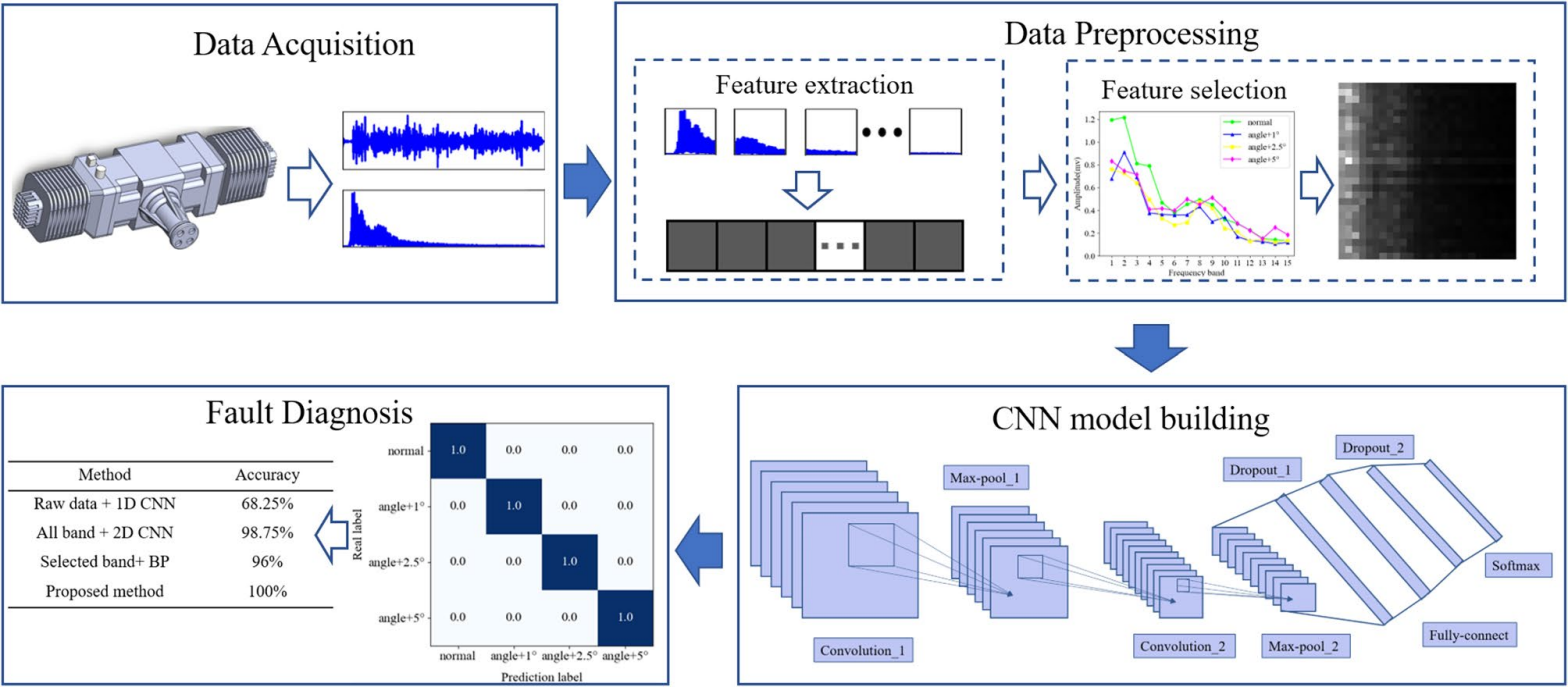
The proposed framework for abnormal identification of ignition advance angle.

The detailed process of identifying the abnormality of the ignition advance angle of the piston aero-engine in this paper is shown in Fig. 2, which mainly includes 4 steps. Firstly, the method of AE data acquisition is introduced, including the selection of AE sensor measurement points and the setting of different states of ignition advance angle. The data pre-processing stage is divided into two steps of feature extraction and feature selection. The AE raw signal is converted from the time domain to the frequency domain by FFT, and the spectrum is divided into equal length frequency bands. Extracting the maximum value of amplitude within each band as the feature value of the band. Then the 1-D feature vector is constructed by selecting the frequency band with greater contribution to the classification based on the distance method and finally stacks the 1-D feature vector in time order to form a 2-D feature map. Next, build a CNN model for fault identification. Finally, an experiment is established to verify the effectiveness of the proposed method.

### Data acquisition

Since the abnormality of the ignition advance angle directly affects the combustion process in the engine cylinder, the combustion chamber is closer to the source of the abnormal signal, and the transmission path of the AE signal is shorter. If the measuring point is selected on the surface of the combustion chamber body, the path coupling effect can be avoided, and a high signal-to-noise ratio of the AE signal can be effectively guaranteed. However, the surface of the combustion chamber of the piston aero-engine used in this paper is a sheet-layer heat dissipation structure, and no effective measuring point can be set. Therefore, the AE sensor is finally chosen to be installed on the smooth surface of the crankcase, which is closer to the combustion chamber. At the same time, a coupling agent is used on the surface of the AE sensor and the airframe surface to enhance the transmission ability of the AE wave so as to ensure the integrity of the collected signals. The ignition advance angle is adjusted by ECU when setting different fault types. They are respectively set as a normal state and three abnormal states. Because the ignition advance angle abnormally set too small on the performance of the engine is too small, there is no recognizable value; on the contrary, if it is set too large will lead to the engine to produce knock and thus cause damage to the engine, so according to past experience, this paper will be set in its abnormal state ignition advance angle increased of 1°, increased of 2.5° and increased of 5°.

### Feature extraction

For vibration sensors, the time domain data of the acquired raw signal is often directly used as the input of the deep learning model^32,33^. However, the AE sensor used in this paper has a high sampling frequency and the amount of acquired data is very large. If the raw time series data is directly input into the CNN model, it will lead to a huge network structure, which will not only increase the calculation time and consume computing resources, but also cause serious overfitting phenomenon. If the classic time-domain features are extracted as the input of the model, although the calculation amount can be reduced, the recognition accuracy cannot be guaranteed due to the large loss of original data information. In addition, since it is difficult to separate noise or useful information from time domain signals, the noise information contained in it will also have a certain impact on the recognition accuracy of the model. Therefore, for the AE signal in this paper, the time domain information is not suitable to be directly used as the input of the CNN model. However, the Fast Fourier Transform (FFT) can effectively denoise the raw signal, and the obtained spectrogram can directly reflect the information of each frequency component of the signal. So this paper uses frequency domain information to identify the abnormality of engine ignition advance angle.

Different from the traditional frequency domain feature extraction method, this paper divides the spectral delineation of the AE signal into m equal-length bands in each cycle, and since the higher amplitude in the spectrum represents the higher energy distribution, the maximum value of the amplitude within each band is extracted as a feature of the band^34^, which aims to characterize the AE signal efficiently while significantly reducing the dimensionality of the data. The set of these m feature values is the eigenvector T, then the eigenvector T of the tth cycle signal can be expressed as:

1 $$\begin{array}{c}T=\left\{max\left({e}_{1}\right),max\left({e}_{2}\right)\dots max\left({e}_{m}\right)\right\},\end{array}$$

where e represents the divided each frequency band.

### Feature selection

Through the above method, m features can be extracted for each cycle signal, but because the amount of information contained in each frequency band is different, especially in some frequency bands, the information displayed by the normal state and abnormal state of the ignition advance angle is almost the same. At this time, we consider that the features of this frequency band have little even no contribution to the identification of the abnormal state of ignition advance angle, which is called redundant features. These redundant features will not only increase the calculation time of the model, but even reduce the recognition accuracy. Therefore, feature selection is required to eliminate useless features. In this paper, a minimum distance-based feature selection method is used from the above-mentioned feature extraction approaches.

**Figure 3:**
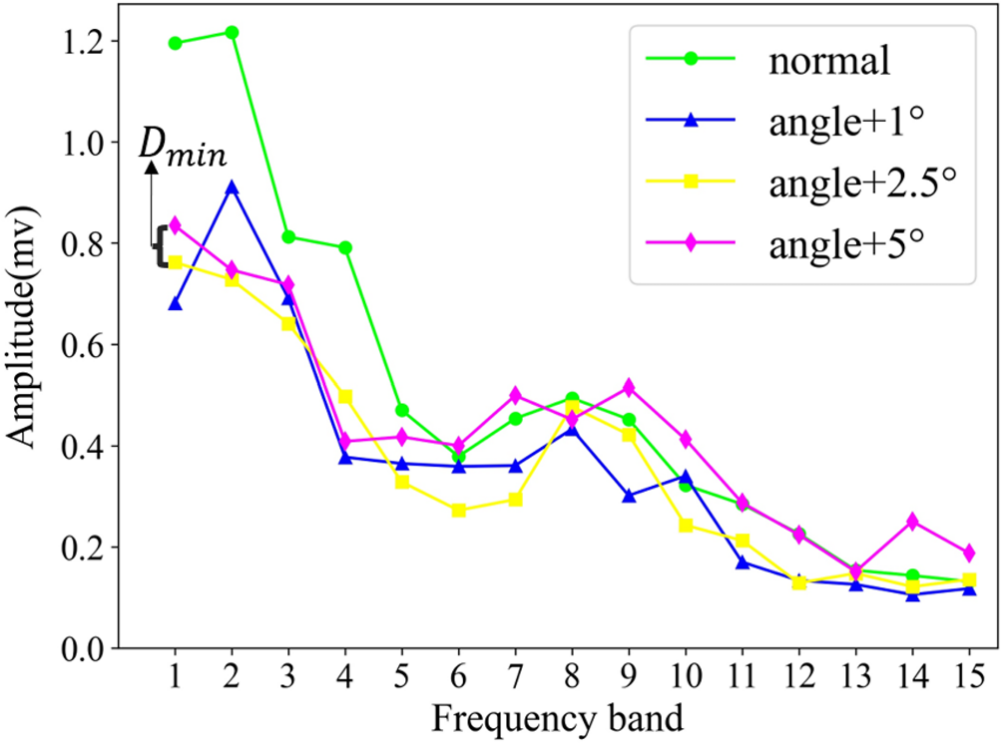
Frequency bands selection.

Suppose ${T}_{1}$, ${T}_{2}$, ${T}_{3}$ and ${T}_{4}$ are the eigenvectors in the four states of ignition advance angle, respectively. The length of the vector is set to 15 (that means the frequency spectrum is divided equally into 15 frequency bands), and their distribution in the line graph is shown in Fig. 3.

It is not difficult to see from the figure that the four fault types have different maximum magnitude values on different frequency bands. In order to select the frequency bands with large information differences and higher contribution to the classification ability, the approach in this paper is to calculate the distance between the maximum magnitude values of these fault types on each frequency band. Based on the concept of minimum differentiation, by comparing the minimum distance in each frequency band as the basis for measuring the classification quality of the frequency band, the larger the minimum distance, the better the discrimination of the frequency band to different fault types. The specific method is as follows:

2 $${({D}_{min})}_{i}=min\left\{\left|{T}_{1i}-{T}_{2i}\right|,\left|{T}_{1i}-{T}_{3i}\right|,\left|{T}_{1i}-{T}_{4i}\right|\right., \left.\left|{T}_{2i}-{T}_{3i}\right|,\left|{T}_{2i}-{T}_{4i}\right|,\left|{T}_{3i}-{T}_{4i}\right|\right\},\left(i=\mathrm{1,2}\dots m\right).$$

From above, the minimum distance of each frequency band can be obtained, and assume M is the mean value of the minimum distance on these m frequency bands:

3 $$\begin{array}{c}M=mean\left\{{\left({D}_{min}\right)}_{1},{\left({D}_{min}\right)}_{2},\dots ,{\left({D}_{min}\right)}_{m}\right\}.\end{array}$$

The mean value M is defined as the threshold value for selecting the frequency band, that is, when the ${({D}_{min})}_{i}$ on the frequency band i is greater than or equal to M, the frequency band is considered as an effective frequency band for fault classification and will be selected; otherwise, when the ${({D}_{min})}_{i}$ on the frequency band i which is less than M, it is considered that the frequency band has little effect on classification and will be discarded. This feature selection method is concise and easy to understand with less computation compared to traditional methods, and significantly improves the recognition efficiency while ensuring high classification ability of the selected features. Through this method, the effective frequency band to distinguish the fault type of the tth cycle signal can be finally selected. Similarly, we can use this method to obtain the effective frequency bands selected by all cycles signal, and finally count the number of times each frequency band is selected. A lower limit is set, and the frequency bands whose selection times are lower than the lower limit will be directly eliminated, and the remaining frequency bands will be used as the final frequency bands for classification.

**Figure 4:**
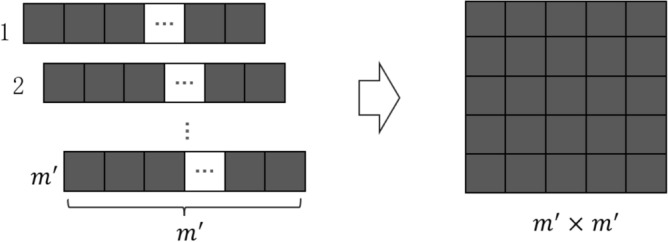
Structural feature map.

Assuming that ${m}^{\prime}$ frequency bands are finally selected, each cycle signal can get its feature vector ${T}^{\prime}$. Next, we need to use the feature vector to construct a feature map as the input of the CNN model. Firstly, the eigenvalues of each cycle signal are mapped to a value between [0,1] using the minimum–maximum normalization method, and then multiplied by 255 to become a gray value within 0 to 255. The specific process is as follows:

4 $$\begin{array}{c}P\left(j\right)=\frac{{{T}^{\prime}}_{1r}-\mathrm{max}\left({{T}^{\prime}}_{1}\right)}{\mathrm{max}\left({{T}^{\prime}}_{1}\right)-\mathrm{min}\left({{T}^{\prime}}_{1}\right)}\times 255,\end{array}$$

where $r=\mathrm{1,2}\dots {m}^{\prime}$. After the above process, at this time each cycle signal can be converted into a 1-D gray value feature vector with a shape of $1\times {m}^{\prime}$. Then, the ${m}^{\prime}$ feature vectors in continuous time are vertically stacked to obtain a 2-D grayscale feature map with a shape of ${m}^{\prime}\times {m}^{\prime}$, the feature map is used as an input to the subsequent CNN model in the last. The process is shown in Fig. 4. Each column of the feature map represents the same frequency band selected in the successive cycles of the signal, while each row represents the eigenvalue within the selected band. The above process makes the ignition advance angle fault feature information more obvious, which will be more beneficial for the CNN model to recognize.

### CNN model construction

**Figure 5:**
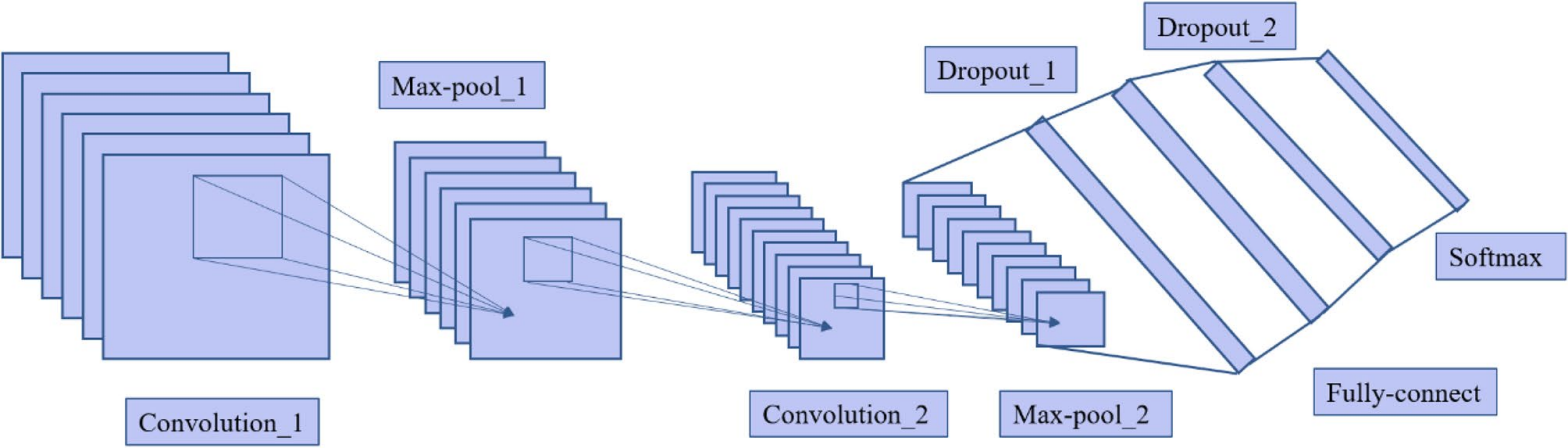
CNN structure diagram.

The CNN model is a typical feed-forward neural network. The essence is to gradually extract the abstract feature representation of the input data in the process of alternating convolution and pooling based on the principle of several filters, and finally classify it by the classification layer. The CNN structure usually contains multiple levels such as input, convolution, pooling, full connected, and output, in which the dropout layer is often added to prevent overfitting to improve the generalization ability of the model, as shown in Fig. 5.

The convolutional layer is the core module of CNN to extract features from input data. The convolutional layer operation performs a convolution operation on the input data through the convolution kernel, and the result of the convolution is the extracted feature. The process of convolution operation is shown in Eq. (5):

5 $$\begin{array}{c}{x}_{j}^{l}=f\left(\sum_{i\in {M}_{j}}{x}_{i}^{l-1}\times {K}_{ij}^{l}+{b}_{j}^{l}\right),\end{array}$$

where ${M}_{j}$ represents the input data set, through the input feature map ${x}_{i}^{l-1}$ of the l layer and the convolution kernel represented by K, the features of the enhanced signal data are convolved, and the output ${x}_{j}^{l}$ of the l layer is obtained, b is the bias value, f represents the nonlinear activation function, and the relu function is generally used.

The pooling layer is usually connected after the convolutional layer, and the input feature map is down-sampled by the pooling kernel to extract features while achieving data dimensionality reduction. Equation (6) shows that after performing the pooling operation down(∙), the weight β and b are added to the reduced feature map to obtain the output of the pooling layer:

6 $$\begin{array}{c}{x}_{j}^{l}=f\left({\beta }_{j}^{l}down\left({x}_{i}^{l-1}\right)+{b}_{j}^{l}\right).\end{array}$$

The fully connected layer converts the 2-D feature map output by the pooling layer into a 1-D feature vector, which plays the role of data dimensionality reduction, and finally feeds the result to the final classifier, which usually uses a softmax classifier to achieve the multi-classification task.

## Experimental verification

### Experiment description

**Figure 6:**
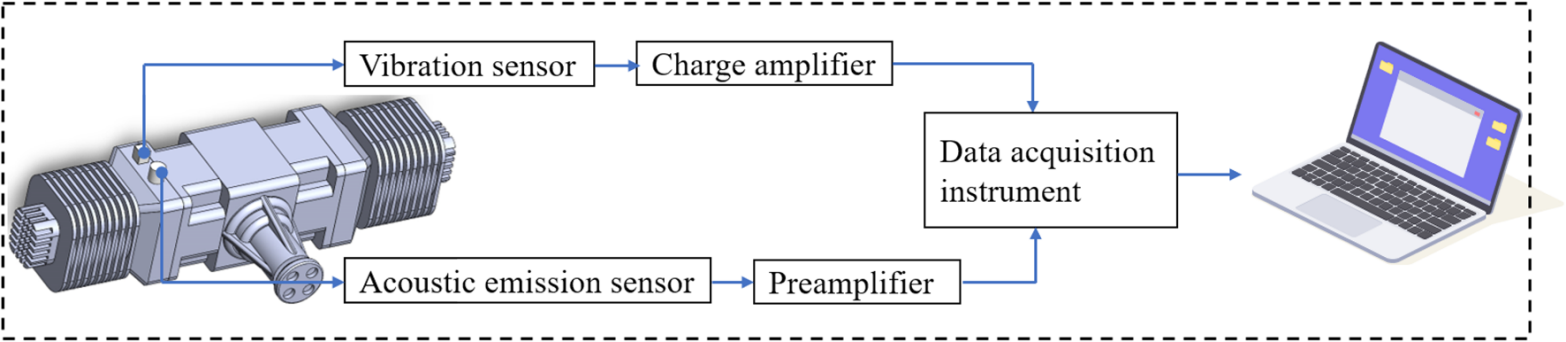
Experimental system diagram.

Figure 6 shows the schematic diagram of the experimental system. The signal acquisition system is composed of AE sensor, preamplifier, data acquisition card and PC. The AE sensor is installed at the position of the 1-cylinder crankcase, the signal-to-noise ratio is improved by using a preamplifier, after which the data acquisition instrument is connected and finally the AE signal is displayed on the PC. In this experiment, the VS45-H AE sensor produced by the Vallen Company in Germany is selected, which has a frequency range of 20 kHz to 450 kHz, and the AE sensor sampling rate is set to 2 MHz according to Nyquist’s sampling rule. The signal acquisition software is also the Vallen Control Panel R2022.0809.2 developed by Vallen Germany. In addition, in order to verify the universality of the method proposed in this paper, this experiment covers multiple operating speed ranges and operating conditions, and the operating condition information is shown in Table 1. Among them, the 4000 rpm working condition, which is the normal flight state of the aircraft, is our main research object. For the safety of the engine, we set the sampling duration of the normal state and each abnormal state to 15 s, discard the data of the first 3 s and the last 3 s, and take the data of the middle 9 s for analysis, so as to eliminate the interference caused by the unstable engine state.

**Table 1:** Information of different working conditions.

	Rotating speed (rpm)	Load (%)	Flight status
1	3000 rpm	10	Idle speed
2	4000 rpm	20	Normal speed
3	4500	30	Accelerate

### Data processing

**Figure 7:**
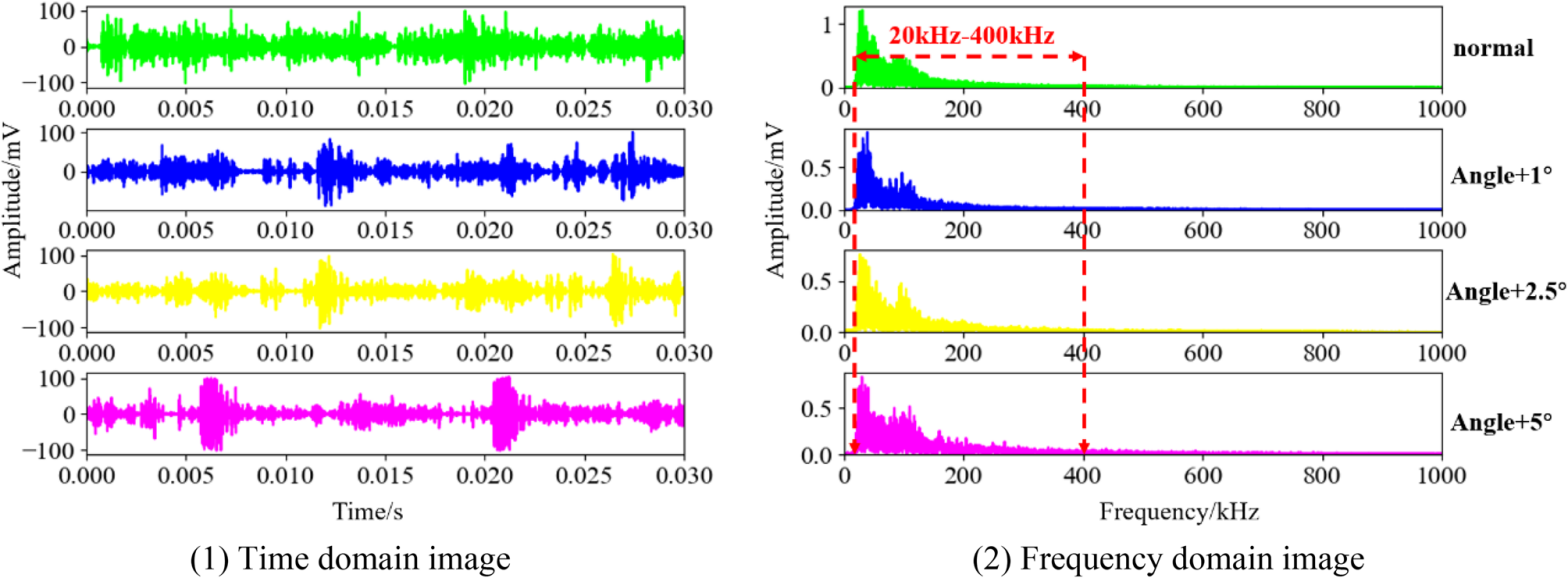
Raw acoustic emission signal.

Since the engine speed is 4000 rpm and the sampling frequency is 2 MHz, each cycle is 0.015 s, which contains 30,000 sampling points. Figure 7(1) is the time domain images in two cycles under the four states of ignition advance angle.

It can be seen from the figure that there is a certain difference in the time domain image of the AE signal under the normal state and abnormal state of the engine ignition advance angle. The signal is smooth in normal state, while the abnormal state has abrupt changes in amplitude, and the abrupt changes become more obvious as the ignition advance angle increases. In order to better judge the working state of the engine and the type of fault, the AE signal is converted from the time domain to the frequency domain through FFT, as shown in Fig. 7(2). From the one-sided spectrum of the frequency domain curve, it is easy to see that the energy gradually shifts to high frequency as the ignition advance angle of the engine increases. Since the frequency range of the selected AE sensor is 20–450 kHz, the information beyond this range is distorted, so we mainly intercept the frequency band of 20–400 kHz for analysis. The frequency band is marked in the figure by the dotted line.

**Figure 8:**
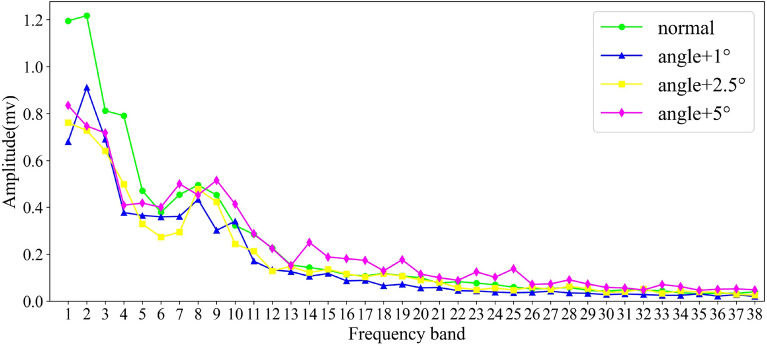
Feature extraction.

Considering calculation efficiency and other issues, the intercepted 20–400 kHz frequency band is equally divided into 38 sub-frequency bands with a length of 10 kHz, and the maximum value of the amplitude is calculated in each frequency band as the feature of this segment, and then the band with high contribution to the classification is selected as the basis for the construction of the feature vector by the method of frequency band selection in “Methodology” section. Figure 8 shows the feature extraction process of a certain cycle in four states of ignition advance angle.

**Figure 9:**
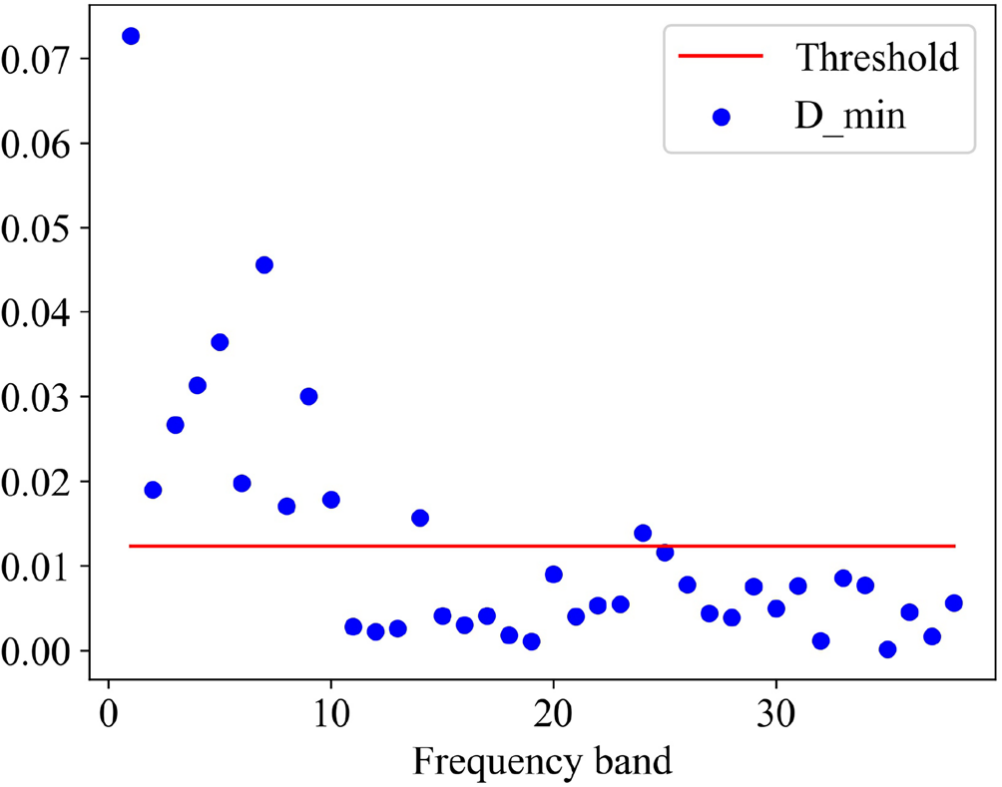
Frequency band selection.

It can be seen from the figure that the features extracted by the ignition advance angle in different states are different in a specific frequency band, and the minimum distance between the four feature values in each frequency band is used as a basis for differentiating the four states of the ignition advance angle. The greater the minimum distance means that the four states are better distinguished. The average value of the minimum distance on these 38 bands is calculated as the threshold value and the frequency band equal to or greater than the threshold value will be selected, and the frequency band smaller than the threshold value is discarded. The red line in Fig. 9 represents the threshold, the blue dots represent the minimum distance on each frequency band, and the frequency band corresponding to the dot above the red line is the selected frequency band, then the serial number of the frequency band selected by this sample are 1, 2, 3, 4, 5, 6, 7, 8, 9, 10, 14, 24.

**Figure 10:**
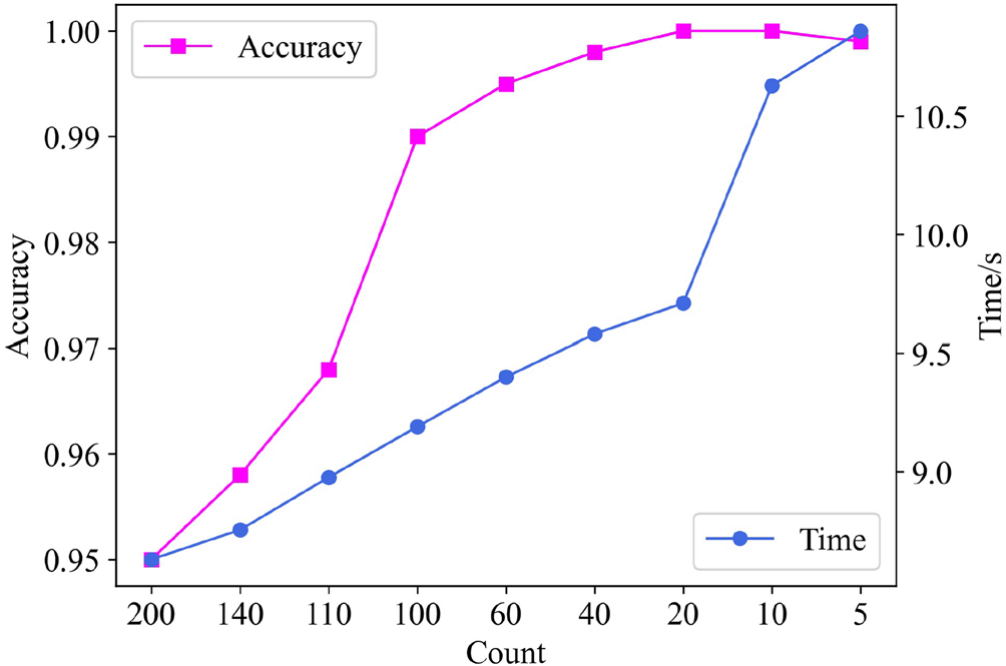
Basis for selection of lower limit.

The above is the process of frequency band selection for a certain cycle, so the frequency bands selected by 400 cycles can be obtained by using the same processing method. Table 2 counts the number of selected frequency bands of 1–38 in 400 cycles, and the order is arranged from high to low. In addition, frequency bands that do not appear in the table mean that the number of times of selection is 0. When determining the lower limit of frequency band selection, we set several nodes according to the statistical counts of each frequency band in Table 2, which are 200, 140, 110, 100, 60, 40, 20, 10, 5. We make each node as a candidate for the lower limit, and calculate the recognition accuracy and recognition time under each node one by one, and the calculation results are shown in Fig. 10. From the figure, it is easy to see that the comprehensive model recognition accuracy and recognition efficiency of both considerations, in the node 20 when the best results, so the lower limit of this paper is set to 20. On account of the number of occurrences of the 1st-26th frequency band exceeds the lower limit, and the other bands are lower than the lower limit, so the 1st-26th frequency band is our final selection.

**Table 2:** Occurrence statistics for each frequency band.

Frequency band	2	1	3	9	8	4	10	7	6
Count	330	329	303	280	276	270	269	257	248
Frequency band	5	11	12	14	15	13	18	17	16
Count	247	226	169	143	139	114	113	106	105
Frequency band	20	19	21	22	23	24	25	26	28
Count	84	70	63	43	40	27	27	27	12
Frequency band	27	29	31	30	32	35	34	33	
Count	10	7	6	6	4	3	1	1

**Figure 11:**
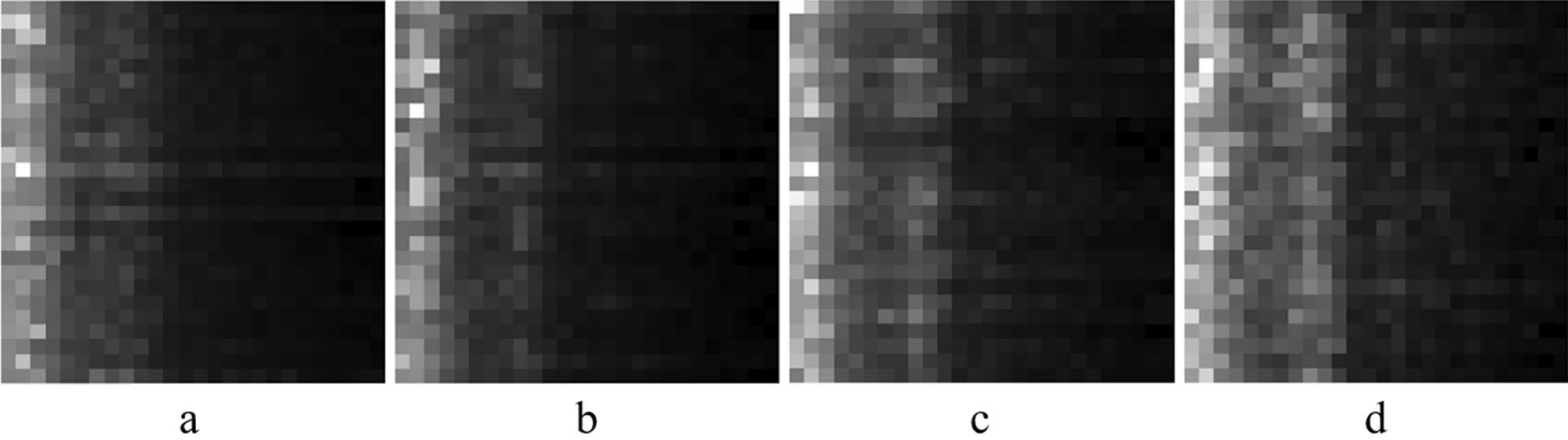
Feature map: (**a**) normal state; (**b**) advance angle + 1°; (**c**) advance angle + 2.5°; (**d**) advance angle + 5°.

The cycle signal generates 1-D feature vectors based on the above-mentioned frequency band selection results, and vertically stacks the feature vectors at continuous moments of the same state of ignition advance angle to construct a 2-D feature map with a shape of 26 × 26. It is easy to see from Fig. 11 that there are obvious differences in the feature maps of the normal state of ignition advance angle and the three abnormal states, which is due to the above data pre-processing process makes the fault feature information more obvious, which will be more beneficial to the subsequent CNN model identification.

### Diagnostic results

The dataset is divided into training set and test set in the ratio of 4:1, with 400 samples for the training set and 100 samples for the test. Table 3 shows the specific structure of the CNN used. It contains three alternating convolutional layers and pooling layers, two fully connected layers and a classification layer. In addition, a dropout layer is added as appropriate to prevent network overfitting. The CNN model is written in Tensorflow 2.7 based on python 3.9. The computer is Windows 10 system with 16 GB RAM, GT 1030 graphics card and Intel i5 processor.

**Table 3:** CNN structure.

Number	Layer type	Layer size
L1	Convolutional layer	3 × 3 × 16
L2	MaxPooling	2 × 2
L3	Convolutional layer	3 × 3 × 32
L4	MaxPooling	2 × 2
L5	Convolutional layer	2 × 2 × 64
L6	MaxPooling	2 × 2
L7	Flatten	576
L8	Full-connect	128
L9	Softmax	4

**Figure 12:**
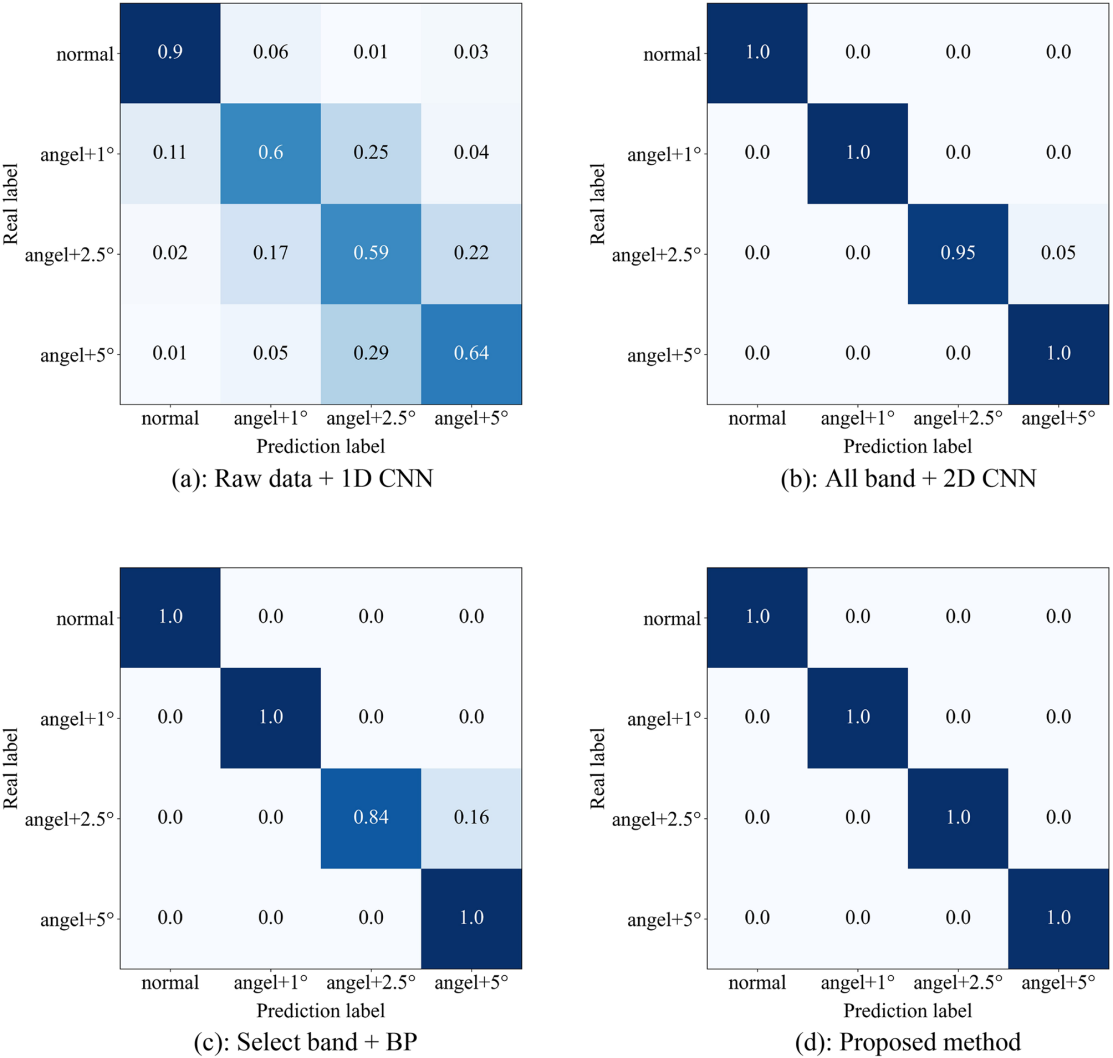
Confusion matrix of test set.

In addition to the proposed method, three other methods will be introduced in this paper to identify the abnormality of engine ignition advance angle. They are: 1. directly input the raw time domain data into CNN for recognition (Raw data + 1D CNN); 2. the equally divided frequency bands are directly used to construct feature maps for input to CNN without selection (All band + 2D CNN); 3. the equally divided frequency band is selected and used to construct a feature map for input to the BP neural network (Select band + BP). Figure 12 shows the confusion matrix of the test set when classification is performed using the four methods described above.

It is not difficult to see from the confusion matrix that the proposed method has the best effect in identifying the abnormality of engine ignition advance angle. In identifying the advance angle + 2.5°, the other three methods except the proposed method all have a certain probability of misidentifying it as the advance angle + 5°. Table 4 compares the recognition accuracy as well as network training time and time per prediction (batch size and epoch are consistent). In order to avoid the chance of a single result, all the data in the table are the average value calculated after running five times. Obviously, among the four methods, method 1 has the lowest recognition accuracy and the longest training time for the network and the longest time per prediction, which is due to the huge amount of AE data and overfitting in the training process, resulting in poor generalization ability of the final model, thus it can be shown that it is more reasonable to use frequency domain information for identification than time domain information in this paper; the recognition accuracy of method 2 is slightly lower than the proposed method but the training time and prediction time are significantly higher, which shows the necessity of frequency band selection. By selecting the frequency band, the redundant features are eliminated and the recognition accuracy and diagnosis efficiency are significantly improved. The proposed method and method 3 do the same data preprocessing, the difference is that the former uses CNN as the classifier, and the latter uses BP neural network as the classifier. Although the time of the latter has a slight advantage, the recognition accuracy is much lower than the former. In summary, the method proposed in this paper significantly shortens the diagnosis time while ensuring high accuracy.

**Table 4:** Comparison of recognition results.

	Method	Prediction accuracy (%)	Training time (s)	Time per prediction (ms)
1	Raw data + 1D CNN	68.25	37.68	41.2
2	All band + 2D CNN	98.75	12.37	37.3
3	Selected band + BP	96	7.21	31.6
4	Proposed method	100	9.71	32.6

**Figure 13:**
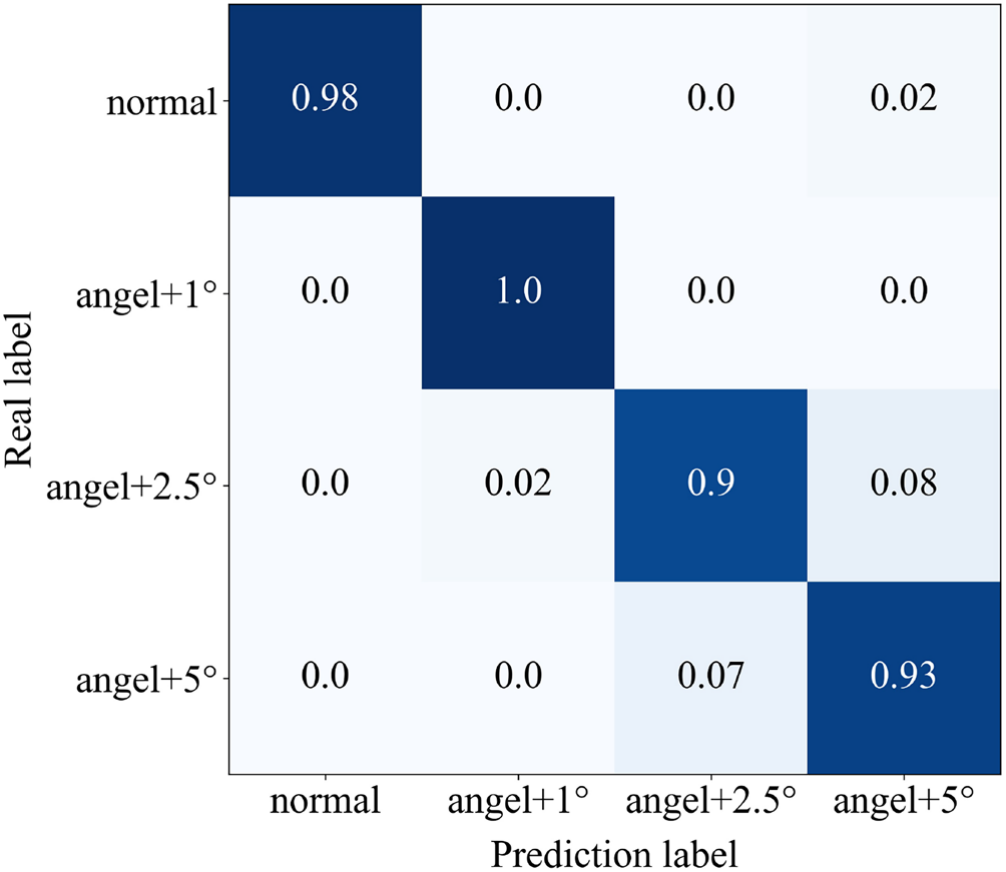
Vibration signal diagnosis results.

In order to illustrate the superiority of the AE signal in identifying the abnormality of the engine ignition advance angle, this paper introduces the vibration signal for comparison. The sampling rate of the vibration sensor is 25.6 kHz, and the same method is used to preprocess the vibration signal and input it to CNN for recognition, as can be seen from Fig. 13, when the vibration signal is used for diagnosis, except for the ignition advance angle + 1°, misdiagnosis occurs in the other three states, and the overall classification accuracy is 95.3%, which is far less than 100% of the AE signal. Therefore, it is advantageously proved that using the AE signal can better identify the abnormality of the engine ignition advance angle than using the vibration signal.

**Figure 14:**
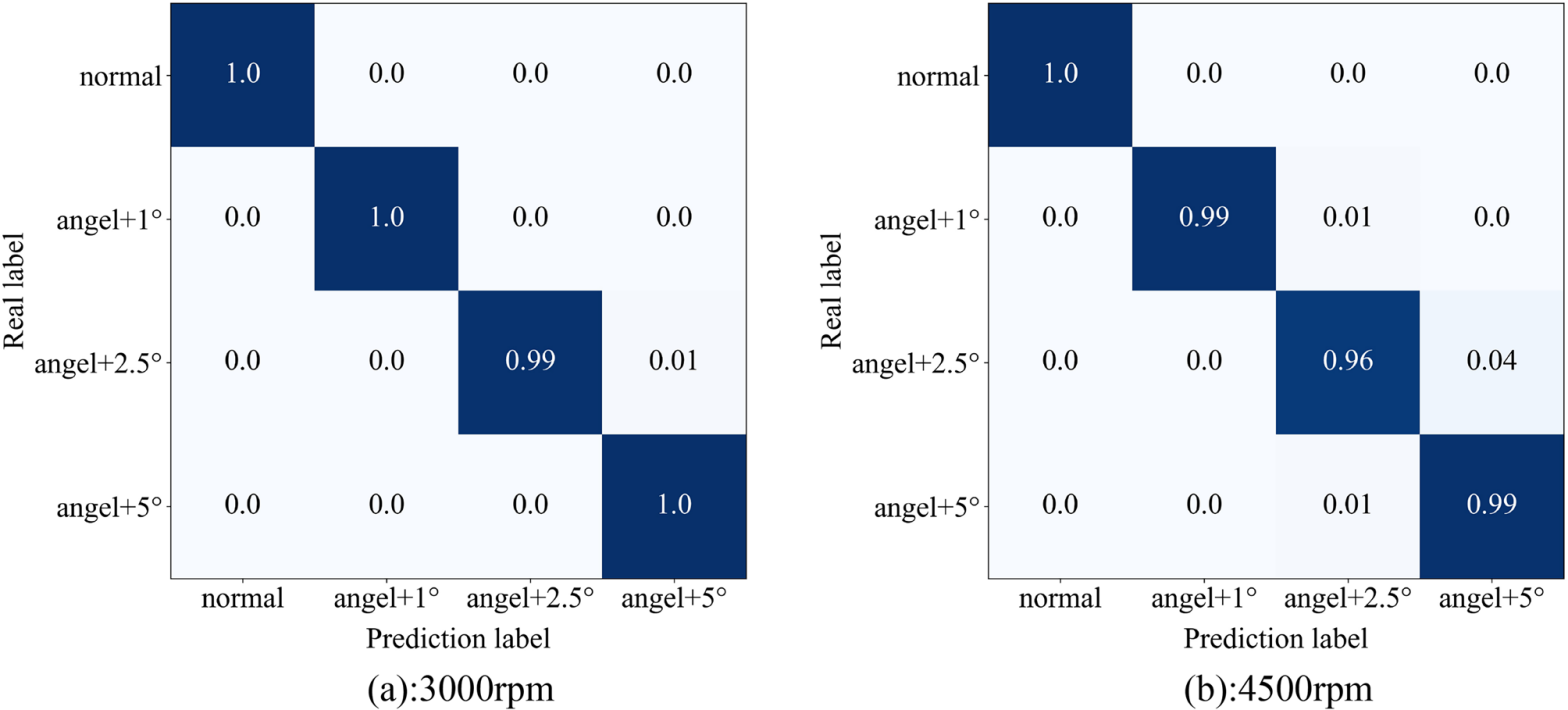
Diagnosis results of different working conditions.

In addition, to verify the universality of the proposed method, we identify the abnormality of the engine ignition advance angle under the two working conditions of 3000 rpm and 4500 rpm respectively. From the classification results in Fig. 14, it can be seen that the proposed method still maintains a high recognition accuracy when applied to these two working conditions, so the method in this paper has a certain degree of universality.

## Conclusion and outlook

This paper proposes an abnormal identification method of ignition advance angle of piston aero-engine based on the combination of AE signal and CNN model. The conclusions obtained are as follows:

- To address the problem of the large amount of AE signal data, which is difficult to directly input into the CNN model, a feature selection method is proposed: select the frequency band with a large contribution to the classification as the basis for the final construction of the feature map, which not only effectively reduces the computational complexity of the model, it also makes the fault characteristic information more obvious, which is more beneficial to fault identification.
- Comparing the proposed method with other conventional methods, the advantages of the former are far greater than the latter in terms of both recognition accuracy and recognition efficiency.
- Comparing the recognition results obtained by using the vibration signal with the recognition results using the AE signal, it is found that the recognition accuracy of the former is far lower than that of the latter, which shows the superiority of using the AE signal to identify the abnormality of the ignition advance angle.
- The proposed method can also maintain high recognition accuracy under other operating conditions of the engine, so the method has a certain degree of universality.

Although the proposed method shows excellent performance, there are still some problems that need to be considered: (a) For reasons of computational efficiency, the frequency band is simply divided into 10 kHz equal lengths in this paper, so the number of band divisions needs to be considered in the next step; (b) in this paper, only one fault type of ignition advance angle is identified, and the proposed method can be used for the identification of multiple fault types in the future.

## Data Availability

This study did not involve any experiments on humans and/or the use of human dataset. The datasets used and/or analysed during the current study available from the corresponding author on reasonable request.
